# Manipulating Propagation Constants of Silver Nanowire Plasmonic Waveguide Modes Using a Dielectric Multilayer Substrate

**DOI:** 10.3390/app8010144

**Published:** 2018-01-22

**Authors:** Yifeng Xiang, Junxue Chen, Douguo Zhang, Ruxue Wang, Yan Kuai, Fengya Lu, Xi Tang, Pei Wang, Hai Ming, Mary Rosenfeld, Ramachandram Badugu, Joseph R. Lakowicz

**Affiliations:** 1 Institute of Photonics, Department of Optics and Optical Engineering, University of Science and Technology of China, Hefei, Anhui 230026, China; 2 School of Science, Southwest University of Science and Technology, Mianyang, Sichuan 621010, China; 3 Center for Fluorescence Spectroscopy, Department of Biochemistry and Molecular Biology, University of Maryland, School of Medicine, Baltimore, Maryland 21201, United States

**Keywords:** Silver nanowire, surface plasmon polariton waveguide, dielectric multilayer, Bloch surface wave, photonic band gap

## Abstract

Experiments and numerical simulations demonstrate that when a silver nanowire is placed on a dielectric multilayer, but not the commonly used bare glass slide, the effective refractive index of the propagating surface plasmons along the silver nanowire can be controlled. Furthermore, by increasing the thickness of the top dielectric layer, longer wavelength light can also propagate along a very thin silver nanowire. In the experiment, the diameter of the silver nanowire can be as thin as 70 nm, with the incident wavelength as long as 640 nm. The principle of this control is analysed from the existence of a photonic band gap and the Bloch surface wave with this dielectric multilayer substrate.

## 1. Introduction

During the last decade, there has been a continuous effort toward the development of plasmonic devices. One reason is surface plasmon polaritons (SPPs), originating from the coupling of an electromagnetic wave to the free electrons of a metal, can strongly confine electromagnetic fields to overcome the optical diffraction limit of dielectric optics, and be very sensitive to the surrounding medium [1–2]. Plasmonic devices are promising to build densely on-chip integrated circuits for next-generation information technology and high performance nanoscale sensors [3–6]. Among plasmonic devices, SPP waveguides are one of the key elements. Different configurations have been proposed and verified and their degree of applicability analysed. For example, a triangular groove milled on a metal surface can work as the channel plasmon subwavelength waveguide [7–8]. Dielectric-loaded SPP waveguides made of a rectangular dielectric rib deposited on a metal film or strip, have recently emerged as a potential plasmonic method that can be integrated seamlessly with current silicon-on-insulator photonic circuits and can sustain transfer of information at high date rates [9–11]. These configurations required top-down nanofabrication. As an alternative, silver nanowires (Ag NWs) synthesized using wet chemistry approaches have proved useful for optical confinement applications below the diffraction limit and for the guiding of light on the nanometre scale, due to their properties of single crystallinity and atomic surface smoothness [12–18]. There are numerous results on SPP waveguides made of Ag NWs. For example, Ag NW SPP waveguides can also serve as highly directional broadband optical antennas, be used to create interferometric logic gates, nanoscale routers and multiplexers, light modulators, and a complete set of Boolean logic functions [19–25]. Ag NW plasmonic waveguides have been integrated with planar polymer optical waveguides for the nanoscale confinement and guiding of light on a chip [26]. The photonic and plasmonic interactions between an individual Ag NW and single-layer MoS_2_ show pronounced nanoscale light–matter interaction between plasmons and atomically thin material that can be exploited for nanophotonic integrated circuits [27]. However, to the best of our knowledge, limited effort has been given to control the propagation constants of the plasmonic mode supported by the Ag NW, which is beneficial for active plasmonic devices. The propagating surface plasmons on supported Ag NWs have been tuned with dielectric (Al_2_O_3_) layers of different thicknesses. This tunability provides a simple and robust way for the precise routing of optical signals in circuitries and new types of ultrasensitive on-chip optical sensors [28]. The current deposition technique can only coat the Ag NW and its substrate (such as glass slide) simultaneously, and thus, the Ag NW will be fixed and cannot be moved to be integrated with other components such as the silicon waveguides. It is not clear that the dielectric layer can be coated uniformly around all the surfaces of the NW. The coating of a dielectric layer may destroy the atomically smooth surface of the single crystal Ag NW, and increase the scattering loss. In this letter, we show that the both the wavenumber (effective refractive index) and propagation distance or cut off wavelength of the SPP waveguide with the Ag NW can be tuned by placement on a dielectric multilayer as the substrate.

## 2. Materials and Methods

**Figure 1:**
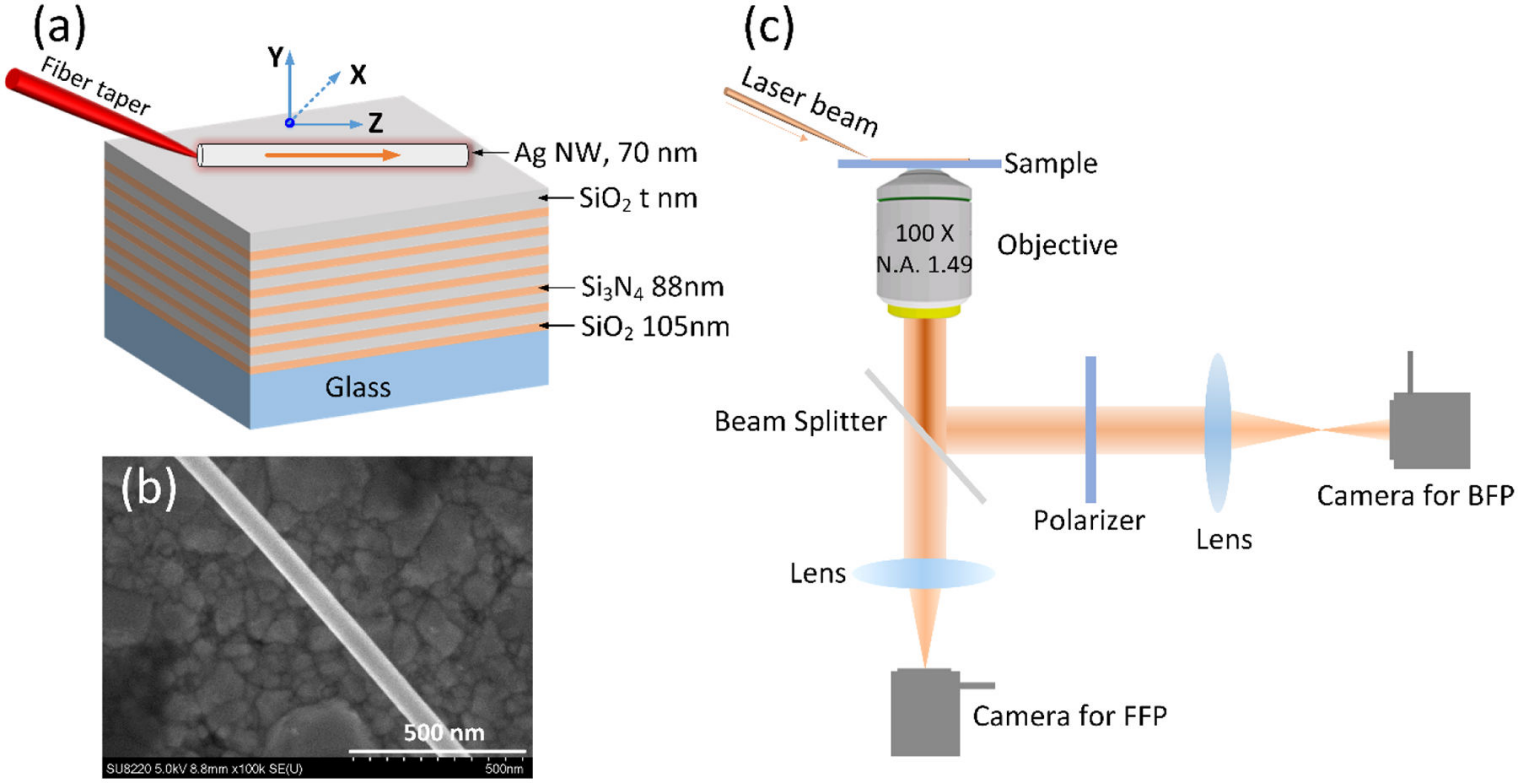
Schematic illustration of the sample. (a) The Ag NW was placed on a dielectric multilayer consisting of alternating layers of SiO_2_ (105-nm-thick) and Si_3_N_4_ (88-nm-thick). There were fourteen layers in total, with a top SiO_2_ layer with a varied thickness of t nm. A fibre taper was used to couple the laser beam into the nanowire. The NW is oriented along the Z-axis and perpendicular to the X-axis. (b) SEM image of the Ag NW (diameter of 70 nm). (c) Schematic of the experimental set-up for imaging the Plasmon’s propagation along the Ag NW. Two cameras are used for capturing the FFP and BFP images. A polarizer can be used to check the polarization state of this Plasmonic mode.

The schematic of the sample is shown in Figure 1(a). The dielectric multilayer (made of alternating SiO_2_ and Si_3_N_4_ layers) was fabricated using plasma-enhanced chemical vapour deposition. The thicknesses of SiO_2_ and Si_3_N_4_ are 105 and 88 nm, respectively. The thickness of the top SiO_2_ layer is defined as t nm, which will be varied in the following experiments and simulations. An Ag NW with a diameter at approximately 70 nm is deposited on this multilayer (Figure 1(b)). The Ag NW was purchased from Nanjing XFNANO Materials Tech Co., Ltd., China. A laser beam (from a SuperK supercontinuum source, NKT Photonics, Denmark) was used for excitation; it was first lens-coupled into a standard single mode silica fibre and its taper would be brought into contact with the Ag NW by a micromanipulator. As a result the laser beam can be coupled into the Ag NW. The beam propagating along this NW was characterized by a home-built instrument for leakage radiation microscopy (LRM). A schematic of the experimental setup (LRM) is shown in Figure 1(c). This LRM can collect both the front focal plane (FFP) and the back focal plane (BFP) images, which can be used to derive the propagation constants (β + i * β’) of the SPP propagating along the Ag NW. The β/K_0_ is the effective refractive index of the plasmonic mode, and the imaginary part (β’) represents the propagation loss. Details of image formation on the FFP and BFP, and how to derive the embedded optical information from the BFP and FFP images, can be found in references [29–33].

## 3. Results

**Figure 2: |:**
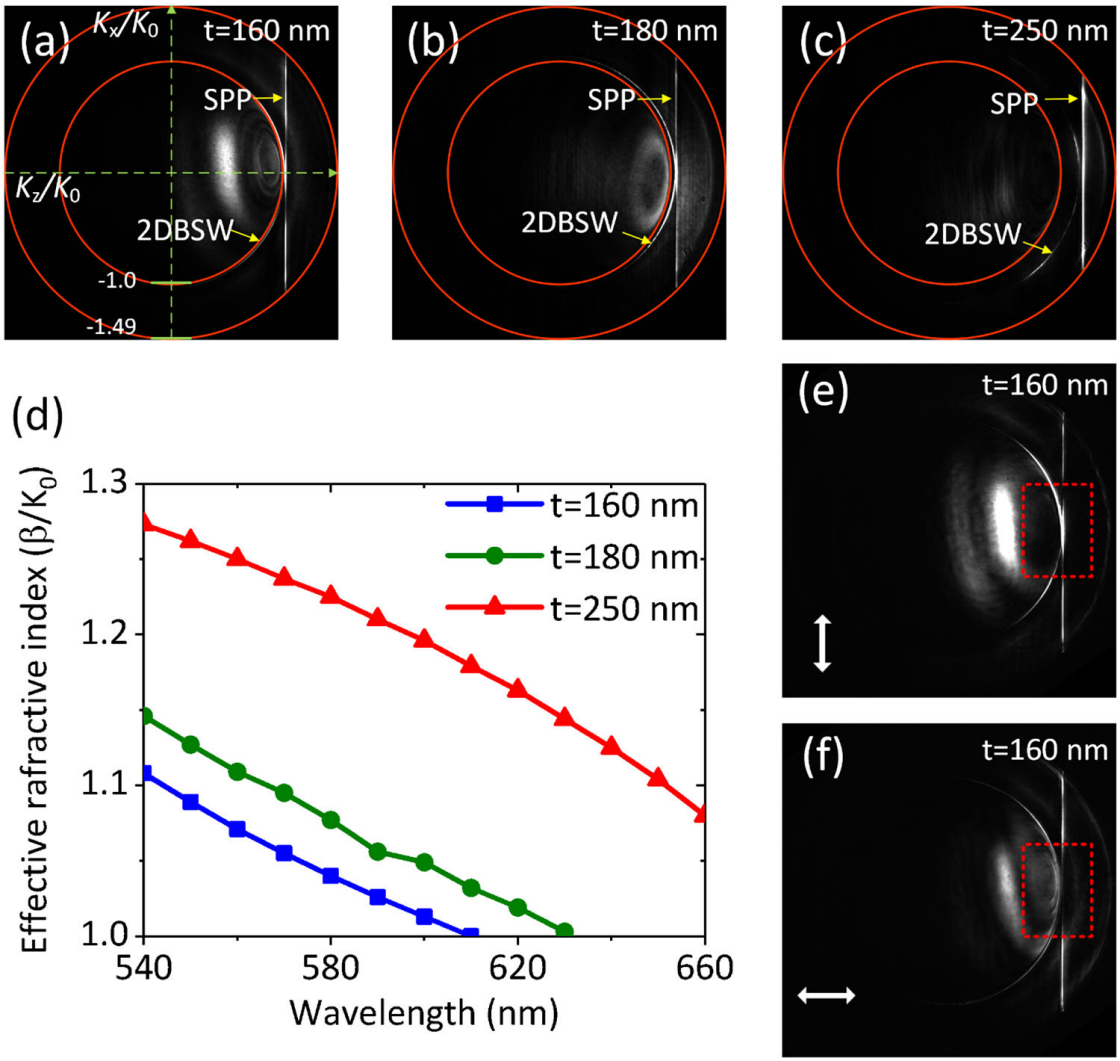
Controlling the effective refractive index of the SPP waveguide mode with the top dielectric (SiO_2_) layer’s thickness. The thickness was selected as t = 160, 180 and 250 nm, respectively. The incident wavelength was selected as 590 nm. (a)-(c), the corresponding BFP images. (d) The curves of effective refractive index vs. the incident wavelength in the case of three top layer’s thickness. (e, f) BFP images in the case of t = 160 nm with a polarizer before the detector. The white double arrowhead lines represent the direction of the polarizer. On (a-c), the red rings with diameters at 1.49 and 1 are given by the NA of the objective and the critical angle, respectively.

In this experiment, three dielectric-multilayer substrates were fabricated, with the top layer thickness (t) selected at about 160 nm, 180 nm and 250 nm. The thickness of the other layers were kept the same as shown in Figure 1(a). By using this dielectric multilayer substrate, SPPs can propagate for a long distance along the thin Ag NW (such as diameter of the Ag NW is less than 100 nm). However, if the same Ag NW was placed on a glass substrate, the propagation distance will be much short [34]. Figure 2(a)–(c) show the BFP images when the Ag NW of 70 nm diameter was placed on these three substrates. The incident wavelength was selected as 590 nm. In our LRM, an oil immersed objective with numerical aperture (NA) at 1.49 was used to collect the leakage plasmonic signals. The outer red rings on Figure 2(a)–(c) represent the largest collection angle that can be derived as arcsin (1.49/1.515)= 79.58 °, where the refractive index of the oil is 1.515. The inner ring represents the critical angle where the total internal reflection occurs, hence the bright areas inside this inner ring are due to the direct transmission of the light through the dielectric multilayer. On the BFP images, the bright vertical line labeled with SPP is the signature of the SPP propagating along the Ag NW [32] and the line is parallel to the axis K_x_/K_0_, which is perpendicular to the NW long axis. From the distance (D) between this vertical line and the axis (K_x_/K_0_), the diameter of the outer ring (R) and known NA (1.49) of the objective, the effective refractive index (or wavenumber, or the real part of the propagation constant) β/K_0_ of this SPP waveguide mode can be calculated with the equation β/K_0_ = (D/R)*NA, where the K_0_ is the wavenumber of the light in vacuum. The SPP propagates along the Z-axis, and the wavenumber along the Z-direction is K_z_/K_0_ =β/K_0_ = 1.03 (a), 1.05 (b) and 1.21 (c), respectively. The BFP images at additional wavelengths ranging from 540 nm to 660 nm were also captured, such as the BFP images at 570 and 610 nm wavelength, which were used to derive out the curves of the effective refractive index vs. incident wavelength, as shown in Figure 2(d). With the increasing of incident wavelength, the effective index decreases, and at all these wavelengths, the effective index will be increased if the top layer thickness is increased from 160 nm to 250 nm. These results clearly demonstrate that the effective index of SPP waveguides mode can be tuned with the thickness (t) of the top SiO_2_ layer (Figure 1(a)).

There are bright arcs on the BFP images (labeled with 2DBSW) appearing closed to the bright lines, which are the two dimensional BSWs (2DBSW) generated by the light from fibre taper. The 2DBSW is the two dimensional surface wave spreading on the surface of the dielectric multilayer. To test the polarization of these modes (2DBSW and of the NW), a linear polarizer was placed before the camera for BFP images, and the corresponding images with two orientations of the polarizer were shown in Figure 2(e) and (f). These two images (the area marked with the dotted box) show that the 2DBSW is of TE polarization, meaning that the polarization direction of the light on the bright arc is perpendicular to the radial direction of the arc, similar as the azimuthal polarization of a vortex beam [35]. For the vertical bright line, the light spot on this line with K_x_/K_0_=0 is dark in the case of horizontal polarization (Figure 2(f)) and is bright in the case of perpendicular polarization (Figure 2(e)), this phenomena reveals that the polarization direction of this light spot is along this bright line, therefore this mode is the H1X mode [36].

**Figure3: |:**
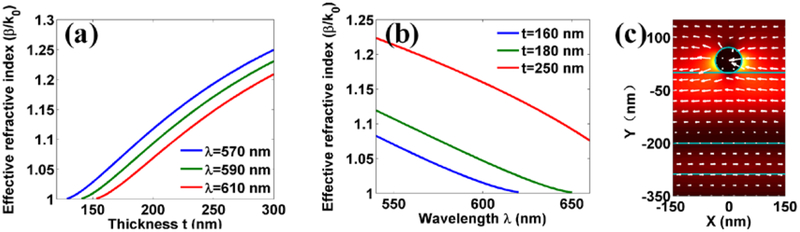
Simulated effective refractive index of the SPP waveguide mode. (a) The effective refractive index of BSW mode versus the thickness of top SiO_2_ layer, in the case of three selected incident wavelength (570, 590 and 610 nm). (b) The effective refractive index of BSW mode versus the incident wavelength with three different thickness of top SiO_2_ layer. (c) The electric field distribution of H1X mode with thickness t = 200 nm and wavelength λ= 590 nm. The arrows with white color denote the directions of the electric fields which shows the mode is the H1X mode. The diameter of Ag nanowire is 70 nm.

Numerical simulations using the finite element method were also carried out to demonstrate the effect of top layer thickness and wavelengths on the effective refractive index. The permittivity of Ag NWs at different wavelengths were based on experimental values [37]. Due to the surface scattering and grain boundary effects in real thin films in the simulations, the refractive indices of SiO_2_ and Si_3_N_4_ are n_SiO2_ = 1.46 + i10^−3^ and n_Si3N4_ = 2.14 + i5 × 10^−3^, respectively. The refractive index of the glass substrate is n_glass_ = 1.515. The thickness of each dielectric layer is the same as that shown in Figure 1(a). Figure 3(a) demonstrates the calculated effective refractive index of plasmonic H1X mode as a function of the top layer thicknesses with three incident wavelengths (570, 590 and 610 nm, the identical to those used on the BFP images in Figure 2). The diameter of the nanowire is 70 nm. The simulation results show that the effective refractive index (β/k_0_) of H1X mode is decreased with the decreasing of top layer thickness, which is consistent with the results derived from the BFP images on Figure 2(a–c). As the wavelength is decreased, the cutoff thickness of H1X mode is also decreased. For example, when the incident wavelength is 610 nm, this mode disappears as the top layer thickness is less than 160 nm. Whereas, at 570 nm wavelength, the same mode disappears at a top layer thickness of 130 nm. To understand the wave guide behavior of H1X mode placed on the dielectric multilayer, the electric field distribution of H1X mode was calculated and shown in Figure 3(c). Different from the case of nanowire placed on the glass substrate, the predominately electric field energy of H1X mode of nanowire is localized near the top layer of dielectric multilayer. The changes of the top layer thickness can adjust the field localization of H1X mode, and greatly affect the wave guiding behavior of this H1X mode. Figure 3(b) shows the effective refractive index of H1X mode at three different top layer’s thickness (t = 160 nm, 180 nm and 250 nm, the same as those used in the experiment) as a function of the incident wavelength. As expected, the change of the effective refractive index with the incident wavelength is consistent with the experimental results shown in Figure 2(d). At all three thicknesses, the index decreased with the increasing of the incident wavelength from 540 nm to 660 nm.

**Figure 4: |:**
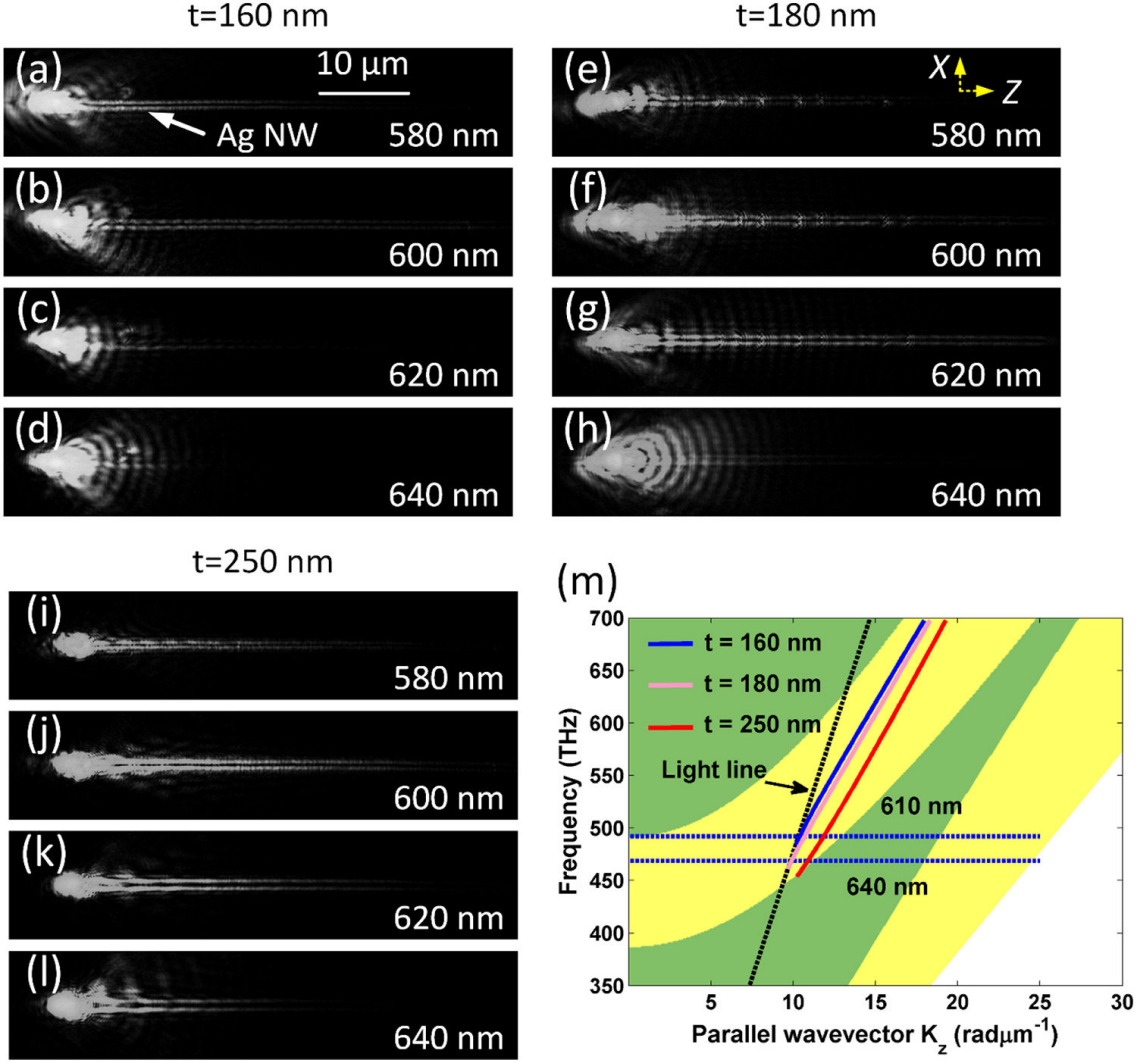
Controlling the propagation distance of the SPP waveguide mode with the top SiO2 layer’s thickness. (a)- (d) the thickness of the top dielectric layer is t = 160 nm, (e) - (h) the thickness of the top dielectric layer is t =180 nm, (i)-(l) the thickness of the top dielectric layer is 250 nm. The incident wavelength varied from 580 to 640 nm with 20 nm step as labelled from (a) to (l). The scale bar in (a) is also applicable for the images in (b)–(l). The dimeter of the Ag NW is about 70 nm on all these images. (m) Simulated band structure of the multilayer and dispersion relation of the SPP waveguide mode. The projected photonic band structure of the dielectric multilayer for TE polarization. The yellow zone denotes the stop band. The solid lines denote the dispersion relations of BSW modes with different thickness of the top SiO_2_ layer (t =160, 180, 250 nm). The diameter of Ag nanowire is 70 nm.

Figure 3(b) also reveals another interesting phenomenon, with increasing the top layer thickness, the cutoff wavelength for this H1X mode will be increased. For example, when the top layer thickness is t = 160 nm, this mode will be cutoff with the incident wavelength longer than 620 nm, whereas, when the thickness is increased into 250 nm, this mode can also appear when the incident wavelength is 660 nm. In our experiment, FFP images of the plasmon propagating along this 70 nm diameter Ag NW at different wavelengths and top layer thicknesses are shown in Figure 4. The selected wavelengths were 580, 600, 620, and 640 nm, respectively. Figure 4(a–d) shows the top layer thickness is approximately 160 nm, and it is clear that at 620 nm wavelength, we can barely find the plasmon propagating along the Ag NW. On the contrary, in Figure 4(i–l), the top layer is at the thickness of approximately 250 nm, and the plasmon can be clearly observed propagating along the NW even if the wavelength is 640 nm. Experiments and simulations verified that the top layer thickness can be used to tune the cut off wavelength of the plasmonic H1X mode on the Ag NW.

To further confirm the mechanism of the above phenomena, the projected photonic band structure of the dielectric multilayer for Transverse electronic (TE) polarization was simulated as shown in Figure 4(m) [38]. The dispersion relations of H1X mode at the three top layer’s thicknesses were also plotted in this figure. The three curves (t = 160 nm, 180 nm and 250 nm) of the dispersive (dispersion) relations all located inside the stop band gap (the yellow zone on Figure 4(m)) of this multilayer and lie on the right side of the light line, which is the fundamental reason that the plasmons can be confined and propagate along this very thin Ag NW. As shown in Figure 4 (m), we can locate the cutoff wavelength for the plasmonic mode decreases with the decreasing of the top layer thickness. This phenomenon also can be analyzed from the change of effective index of the plasmonic mode with the incident wavelength and top layer thickness. As shown in Figures 2 and 3, in the case of thin top dielectric layer, such as t =160 nm, its refractive index is smaller than that on a thicker one (t = 250 nm), and with the increasing of the incident wavelength, the refractive index of this SPP mode will decrease accordingly. When this index is approaching 1.0, this mode will be cut off. It is easy to understand that SPP mode on the top layer with t =160 nm has a shorter cutoff incident wavelength.

**Figure 5|:**
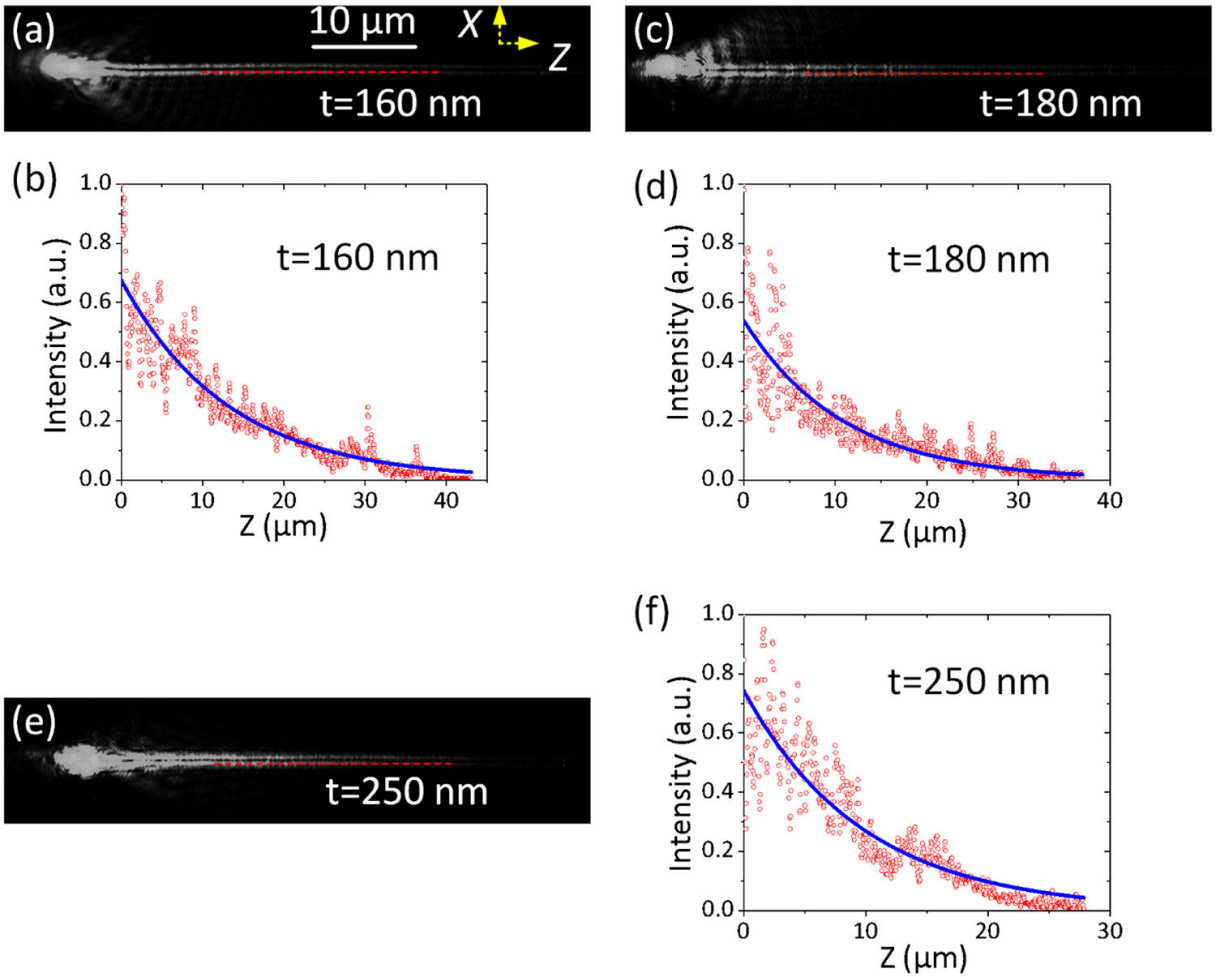
FFP images of the Plasmon’s propagations. Three Ag NWs of the same diameter (about 70 nm) were put on three substrates with different thickness of the top SiO_2_ layer. The top SiO_2_ layer’s thickness is t = 160 nm (a-b), t =180 nm (c-d), and t= 250 nm (e, f), respectively. The incident wavelength is selected as 590 nm. On (b, d, and f), the blue solid line is an exponential fit to the data (red dots) and was used to extract the propagation distance of the plasmonic mode as 13 μm (at t =160 nm), 11 μm (at t =180 nm), and 10 μm (at t = 250 nm). The scale bar in (a) is also applicable for (c, e)

It should be noted that the imaginary part (β’) of the propagation constant represents the propagation distance of this plasmonic mode, and from Figure 4, it is evident that the top dielectric layers affect the propagation distance. As an example, the propagation distances of the SPPs along the Ag NW placed on three multilayer substrates have been fitted and derived as shown in Figure 5. The incident wavelength is fixed at 590 nm, and the thickness of the top SiO2 layer is selected as t= 160 nm, t= 180 nm, and t = 250 nm, respectively. The thickness of the other 13 layers were kept the same as shown in Figure 1 (a). It is clearly showed that, although the incident wavelength and diameter of the Ag NW are both the same on the three multilayer substrates, the propagation distances are different from each other. The propagation distances of the plasmonic mode were derived as 13 μm (at t =160 nm), 11 μm (at t =180 nm), and 10 μm (at t = 250 nm), respectively.

## 4. Conclusions

In conclusion, a new method has been proposed to mediate the propagation constant of the SPP mode on a very thin Ag NW (diameter as low as 70 nm), when placed on a dielectric multilayer. Both the effective refractive index and propagation distance or cut off wavelength of this mode can be tuned with thickness of the top dielectric (SiO_2_) layer. When the Ag NW of the same diameter was placed on a commonly used glass or silicon substrate, the SPP mode here (H1X mode or plasmonic leaky mode) will disappear, and only the plasmonic bound mode can remain but with very short propagation distance [34]. With this method, the Ag NW can be placed on any location of the substrate and can be moved flexibly to be integrated with other components. There is no need to coat another surface cladding and will avoid damaging the smooth NW surface. For future applications, the top SiO_2_ layer can be replaced with a layer made of piezoelectric materials, such as LiNbO_3_, whose thickness or refractive index can be tuned with external electric signals providing an approach for realizing active nano-devices. This tuning of the effective refractive index and propagation distance of the SPP waveguide modes on an Ag NW, can be used to understand a serial of Boolean logic functions in on-chip integrated circuits for next-generation information technology and nanoscale optical sensing. We note that the field intensity is stronger confined near the NW surface (Figure 3e). This confinement will result in a small observed volume which is of interest for biophotonic applications [39–42].
